# LncRNA HOTAIRM1 promotes osteogenesis by controlling JNK/AP‐1 signalling‐mediated RUNX2 expression

**DOI:** 10.1111/jcmm.14620

**Published:** 2019-09-11

**Authors:** Lei Fu, Shifang Peng, Wanfeng Wu, Yi Ouyang, Deming Tan, Xiaoyu Fu

**Affiliations:** ^1^ Department of Infectious Diseases, Key Laboratory of Viral Hepatitis Xiangya Hospital, Central South University Changsha China; ^2^ School of the Integrated Traditional Chinese and Western Medicine Hunan University of Chinese Medicine Changsha China

**Keywords:** acetylation, HOTAIRM1, JNK/c‐Jun signalling, osteogenesis, p300

## Abstract

Mesenchymal stem cells (MSCs) have potential ability to differentiate into osteocytes in response to in vitro specific induction. However, the molecular basis underlying this biological process remains largely unclear. In this study, we identify lncRNA HOTAIRM1 as a critical regulator to promote osteogenesis of MSCs. Loss of HOTAIRM1 significantly inhibits the calcium deposition and alkaline phosphatase activity of MSCs. Mechanistically, we find that HOTAIRM1 positively modulates the activity of JNK and c‐Jun, both of which are widely accepted as crucial regulators of osteogenic differentiation. More importantly, c‐Jun is found to be functionally involved in the regulation of RUNX2 expression, a master transcription factor of osteogenesis. In detail, c‐Jun can help recruit the acetyltransferase p300 to RUNX2 promoter, facilitating acetylation of histone 3 at K27 site, therefore epigenetically activating RUNX2 gene transcription. In summary, this study highlights the functional importance of HOTAIRM1 in regulation of osteogenesis, and we characterize HOTAIRM1 as a promising molecular target for bone tissue repair and regeneration.

**Highlights**

- HOTAIRM1 functions as a critical activator to regulate osteogenic differentiation.
- HOTAIRM1 positively regulates the activity of JNK/AP‐1 signalling pathway.
- HOTAIRM1 elevates expression of the osteogenic regulator RUNX2 through a c‐Jun/p300‐mediated acetylation mechanism.

## 1 INTRODUCTION

As is well known, osteoblasts and osteoclasts, the two main cell types of bone, cooperatively regulate the dynamic bone renewal and regeneration.^1^, ^2^, ^3^ However, in some terrible conditions such as osteolytic bone tumour surgery and osteonecrosis, the bone regeneration progress will be disabled or go beyond its self‐repair ability. Therefore, the bone tissue engineering, particularly the stem cell–based therapy, has become a good choice in clinical therapeutics of these dysregulated bone tissue defects.^4^, ^5^, ^6^, ^7^ Mesenchymal stem cells (MSCs), derived from a number of tissues such as adipose and bone marrow, have been found to have potential ability to differentiate into multilineage cells, like osteocytes and adipocytes, upon in vitro specific induction.^8^, ^9^, ^10^, ^11^, ^12^, ^13^ Thus, MSC is a good candidate for the clinical treatment of multiple bone tissue‐regulated defects.

MAPK/JNK signalling is found to be functionally involved in the regulation of osteogenic differentiation of MSCs.^14^, ^15^ Suppression of the JNK signalling significantly reduces the calcium deposition and alkaline phosphatase activity, as well as expressions the representative osteogenic marker genes by an in vitro study. On the other hand, the inactivated JNK signalling can also inhibit the bone formation by using an athymic nude mice model system.^16^ Therefore, study on the regulation of JNK signalling activity is quite important for MSC‐based bone tissue engineering. Moreover, increasing evidence revealed that the osteogenesis of MSCs can be regulated by various osteogenic regulators, especially the runt‐related transcription factor 2 (RUNX2) which is one of the most critical master transcription factors during osteogenic progress. RUNX2 attenuation could repress osteogenic differentiation and some other osteogenic regulators implicated in osteogenic progression.^17^, ^18^, ^19^, ^20^

Long non‐coding RNAs (lncRNAs), which are a subset of non‐coding transcripts with more than 200 nucleotides in length, have been reported to have regulatory roles in numerous cellular disorders, such as inflammation, immunity and various cancers.^21^, ^22^, ^23^, ^24^, ^25^ However, the roles of lncRNAs in osteogenic regulation are poorly defined. In this study, we first identified lncRNA HOTAIRM1 as a crucial regulator of osteogenic differentiation. Knockdown of HOTAIRM1 markedly represses the calcium deposition and alkaline phosphatase activity, suggesting the critical function of HOTAIRM1 in osteogenesis. Moreover, we explored the underlying molecular mechanism and found that HOTAIRM1 mediates the osteogenic regulator RUNX2 expression through a c‐Jun/p300 coordinated acetylation manner, providing a better understanding of the positive regulatory role of HOTAIRM1 in osteogenic differentiation at molecular level.

## 2 MATERIAL AND METHODS

### 2.1 Isolation and culture of MenSCs and UCMSC

The menstrual bloods were collected from the healthy female donors. Equal volume PBS was added to the samples and then subjected to standard Ficoll procedures. Subsequently, the detailed steps for MenSCs isolation and culture were conducted as previously described.^26^ For UCMSC isolation and culture, the umbilical cord vessels were first subjected to disinfection in 75% ethanol for 1 minute and then were cut into cubes. Subsequently, the supernatant was discarded and the precipitates were rinsed with DMEM. In the following, the detailed protocol for UCMSC isolation and culture was conducted as previously reported.^26^

### 2.2 Alizarin Red S staining and ALP activity detection

For Alizarin Red staining, MenSCs were first fixed in 70% ethanol and then subjected to 1% Alizarin Red solution staining for 1 minute. The detailed procedures were performed as previously described.^26^ For the detection of ALP activity, cells were first fixed with 70% ethanol for 30 minutes and then incubated with the BCIP/NBT liquid substrate at 37°C for 30 minutes. The detailed procedures were performed as previously described.^26^

### 2.3 Antibodies and reagents

Anti‐Histone 3 (#17168‐1‐AP) antibody was purchased from Proteintech Group Inc, anti‐p300 (#ab10485) antibody was from Abcam, and anti‐JNK (#9252) and anti‐phospho‐JNK (#4668), anti‐c‐Jun (#9165), anti‐phospho‐c‐Jun (#3270), anti‐RUNX2 (#12556) and H3K27ac (#8173) antibodies were obtained from Cell Signaling Technology. The osteogenic differentiation medium (#SCM121), chemical reagent Bay 11‐7082 (#B5556), Alizarin Red S (#A5533) and BCIP/NBT liquid substrate system (#B1911) were all purchased from Sigma.

### 2.4 Plasmid constructions and lentiviral infection

The shRNA against human HOTAIRM1 was cloned into a modified pLV‐H1‐Puro lentiviral vector. The corresponding sequence for shHOTAIRM1 was 5′‐AATGAAAGATGAACTG GCGAG‐3′. C‐Jun and p300 siRNAs (Thermo Scientific) were transfected by using RNAiMAX transfection reagent (Thermo Scientific). The human HOTAIRM1 was amplified by using reverse transcription PCR and then inserted into a modified pLV‐EF1α lentiviral vector as previously reported.^23^ For lentivirus infection, the detailed experimental procedure was carried out as previously described.^23^

### 2.5 Quantitative RT‐PCR

Total RNAs were extracted from MenSCs or UCMSC using TRIzol reagent, according to manufacturers' instructions. Reverse transcription was performed with 1 μg total RNA. Real‐time quantitative PCR was conducted using an EvaGreen qPCR Master Mix purchased from Applied Biological Materials Inc The relative changes in gene expression were assayed by the 2^−ΔΔCt^ method. The primer sequences used in RT‐qPCR analysis are as follows: for RUNX2 was F. 5′‐GGACGAGGCAAGAGTTTCAC‐3′, R. 5′‐GAGGCGGTC AGAGAACAAAC‐3′; for SP7 was F. 5′‐CACAGCTCTTCTGACTGTCTG‐3′, R. 5′‐CTGGTG AAATGCCTGCATGGAT‐3′; for SPP1 was F. 5′‐AGCCAATGATGAGAGCAATG‐3′, R. 5′‐TCCTTA CTTTTGGGGTCTAC‐3′; for GAPDH was 5′‐F. CATGAGAAGTATGACAACAGCCT‐3′, R. 5′‐AGTCC TTCCACGATACCAAAGT‐3′; for HOTAIRM1 was 5′‐F. CCCACCGTTCAATGAAAG‐3′, R. 5′‐GTTTCAAACACCCA CATTTC‐3′.

### 2.6 High‐throughput mRNA sequencing

The mRNA‐Seq experiment was conducted by Annoroad (Beijing, China). Briefly, total RNAs were extracted using TRIzol reagent and then subjected to library construction according to standard Illumina protocols. The libraries were sequenced with Illumina HigSeq × Ten sequence platform using the paired‐end RNA‐seq approach. For subsequent data analysis, the detailed method was performed as previously reported.^24^ The raw data have been deposited in the Sequence Read Archive (SRA) database with an accession number SRP192509.

### 2.7 Chromatin immunoprecipitation (ChIP)

Briefly, 10^7^ MenSCs were cross‐linked with 1% formaldehyde and then quenched with 125 mmol/L glycine solution. The cells were lysed and the DNAs were sonicated into fragments from 100 to 500 bp with a sonicator. Subsequently, the sonicated lysates were cleared and incubated with indicated antibodies for immunoprecipitation. The immunoprecipitates were reversed, and the DNA was eluted with an elution buffer for subsequent quantification. The primer sequence of ChIP assays for RUNX2 is F. 5′‐ACCATGGTGGAGATCATCG‐3′, R. 5′‐GGCAGGGTCTTGTTGCAG‐3′.

### 2.8 Statistical analysis

Student's *t* test was used to compare two groups. For a comparison of ≥3 groups, one‐way ANOVA followed by Tukey post hoc test was used. *P*‐value <.05 was considered statistically significant. All data are obtained from at least three independent experiments and presented as mean ± SD.

## 3 RESULTS

### 3.1 HOTAIRM1 expression is markedly induced upon osteogenic induction

**Figure 1:**
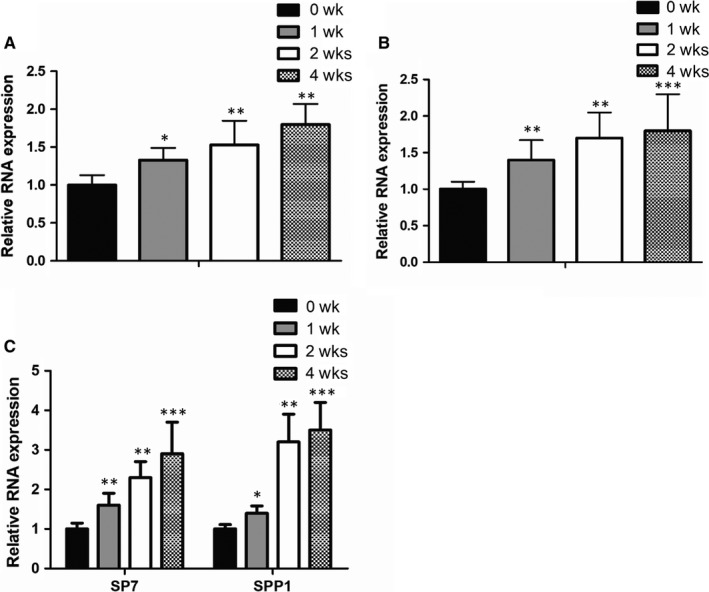
HOTAIRM1 expression was induced after osteogenic medium induction. A, B, RT‐qPCR assays were performed to examine dynamic changes of HOTAIRM1 expression in MenSCs (A) and UCMSC (B) upon osteogenic induction for 1, 2 and 4 wk, respectively. C, Expressions of the representative osteogenic marker genes SP7 and SPP1 in MenSCs with osteogenic medium treatment for 1, 2 and 4 wk, respectively, were assayed by RT‐qPCR analysis. All results are from biological triplicates, and data shown are the mean ± SD. n = 3. *P < .05, **P < .01, ***P < .001 vs 0 wk

As is previously reported, LncRNA HOTAIRM1 can function in diverse physical and pathological processes. However, the regulatory role of HOTAIRM1 in osteogenesis has not yet been discovered. To study the functional involvement of HOTAIRM1 in this biological process, we purchased a commercial medium which is widely used for osteogenic induction, and then used it to treat two types of mesenchymal stem cells to observe the dynamic changes in HOTAIRM1 expression. Notably, expressions of HOTAIRM1 in both menstrual blood‐derived mesenchymal stem cells (MenSCs) and umbilical cord mesenchymal stem cells (UCMSC) were significantly increased after osteogenic medium treatment (Figure 1A,B), indicating the potential regulatory function of HOTAIRM1 during osteogenic differentiation. Meanwhile, we examined expressions of the representative osteogenic marker genes, such as Sp7 transcription factor (SP7) and secreted phosphoprotein 1 (SPP1). As a consequence, expressions of SP7 and SPP1 were dramatically augmented when subjected to osteogenic induction (Figure 1C).

### 3.2 HOTAIRM1 promotes osteogenesis of mesenchymal stem cells

**Figure 2:**
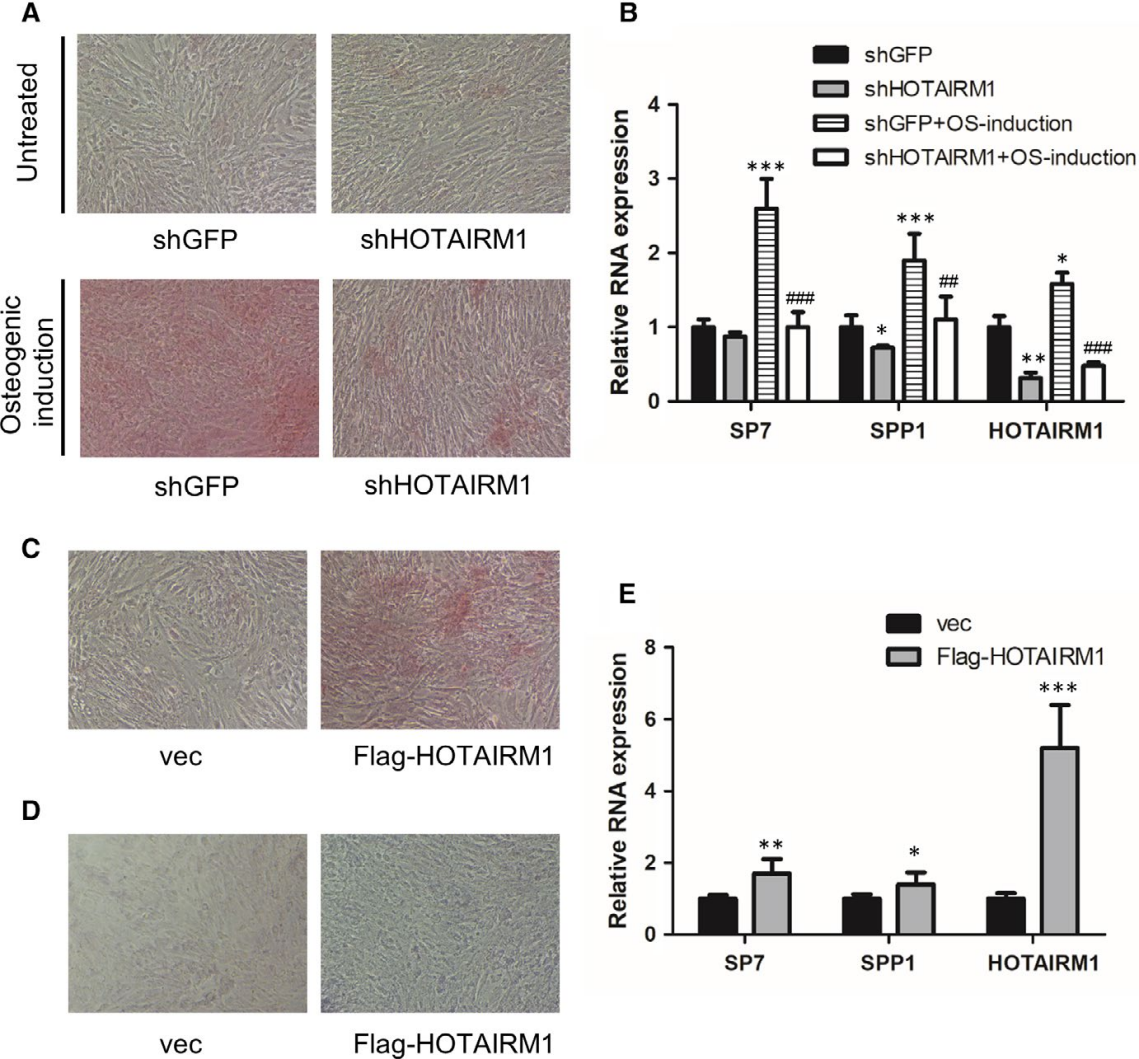
HOTAIRM1 positively regulates the osteogenic differentiation of mesenchymal stem cells. A, The effect of HOTAIRM1 on calcium deposition of MenSCs, with or without osteogenic induction for 3 wk, was determined by Alizarin Red S staining analysis. B, RT‐qPCR assay was conducted in MenSCs in the presence or absence of osteogenic induction for 2 wk with or without HOTAIRM1 knockdown, to measure the effect of HOTAIRM1 on expressions of the osteogenic markers SP7 and SPP1. C, Aberrant HOTAIRM1 overexpression in MenSCs was performed to test the effect of HOTAIRM1 on calcium deposition. D, Aberrant HOTAIRM1 overexpression in UCMSC was performed to examine the effect of HOTAIRM1 on alkaline phosphatase activity. E, Expressions of the osteogenic markers SP7 and SPP1 in UCMSC, with or without HOTAIRM1 overexpression, were detected by RT‐qPCR analysis. All results are from biological triplicates, and data shown are the mean ± SD. n = 3. *P < .05, **P < .01, ***P < .001 vs shGFP or vec; ^##^ *P* < .01, ^###^ *P* < .001 vs shGFP + OS‐induction

To define the regulatory function of HOTAIRM1 in osteogenesis of mesenchymal stem cells, we first performed shRNA‐mediated HOTAIRM1 knockdown in MenSCs and then conducted Alizarin Red S staining to observe the effect of HOTAIRM1 on calcium deposition. Consequently, we found that attenuation of HOTAIRM1 markedly reduced calcium deposition of the MenSCs (Figure 2A). Meanwhile, we determined expressions of the representative osteogenic markers in control and HOTAIRM1‐depleted MenSCs, respectively. RT‐qPCR analysis revealed that HOTAIRM1 depletion markedly reduced expressions of the osteogenesis‐associated markers, like SP7 and SPP1 (Figure 2B). To confirm the regulatory role of HOTAIRM1 in osteogenesis, we next constructed a lentiviral vector of HOTAIRM1 and then performed HOTAIRM1 ectopic overexpression in MenSCs and UCMSC, respectively. As a consequence, both calcium deposition and ALP activity were dramatically enhanced after enforced HOTAIRM1 overexpression (Figure 2C,D). Consistently, expressions of osteogenic marker genes SP7 and SPP1 were obviously augmented by the highly expressed HOTAIRM1 (Figure 2E). Collectively, these observations suggest that HOTAIRM1 plays a critical positive role in the regulation of osteogenesis.

### 3.3 HOTAIRM1 regulates the activity of JNK/AP‐1 signalling pathway

**Figure 3:**
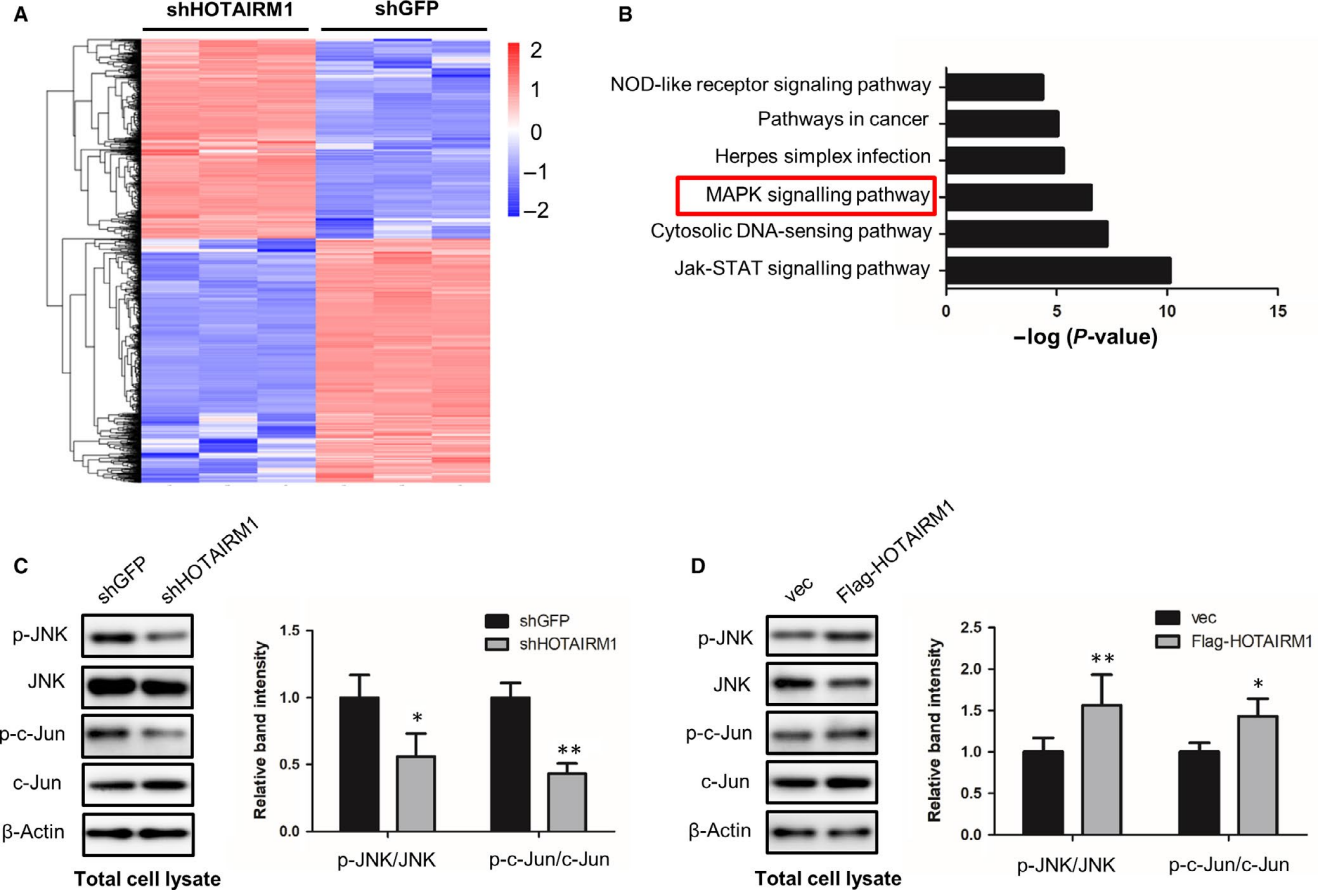
HOTAIRM1 regulates the activity of JNK/c‐Jun pathway. A, Heat map of mRNA‐Seq data in MenSCs showing the differentially expressed genes after HOTAIRM1 knockdown. B, High‐throughput mRNA sequencing was performed in control and HOTAIRM1‐depleted MenSCs, respectively, followed by KEGG pathway analysis to determine HOTAIRM1 downstream‐regulated signalling pathways. C, The effect of HOTAIRM1 on JNK and c‐Jun phosphorylation status was examined in MenSCs with or without HOTAIRM1 knockdown by Western blot assays and then quantified by ImageJ software. D, Lentivirus‐mediated HOTAIRM1 overexpression was conducted in UCMSC, followed by Western blot analysis to show the effect of HOTAIRM1 on the phosphorylation status of JNK and c‐Jun and then quantified by ImageJ software. All results are from biological triplicates, and data shown are the mean ± SD. n = 3. *P < .05, **P < .01 vs shGFP or vec

The above results have shown the crucial role of HOTAIRM1 in osteogenic differentiation. Therefore, we next sought to define the underlying molecular basis by which HOTAIRM1 positively regulates osteogenesis. First, we silenced HOTAIRM1 in MenSCs and then employed high‐throughput mRNA sequencing analysis, to define HOTAIRM1 downstream‐mediated genes and signalling pathways. Of note, 1085 and 1295 genes were found to be significantly up‐ and down‐regulated, respectively, after HOTAIRM1 depletion (Figure 3A). More interestingly, KEGG pathway analysis revealed that HOTAIRM1 is crucially associated with MAPK/JNK pathway which is widely accepted as a critical signalling for osteogenic differentiation (Figure 3B). To validate the influence of HOTAIRM1 on the activity of MAPK/JNK signalling, we detected the phosphorylation status of JNK, as well as its downstream regulator c‐Jun which is the major regulator of AP‐1 transcription factor family, with or without HOTAIRM1 knockdown. Western blot analysis revealed that the phosphorylation levels of JNK at T183 and Y185 sites, as well as c‐Jun at S73 site, were remarkably restrained when HOTAIRM1 was silenced (Figure 3C). Moreover, we conducted HOTAIRM1 enforced overexpression to further confirm the effect of HOTAIRM1 on the activity of JNK signalling. As expected, highly expressed HOTAIRM1 dramatically augmented JNK/AP‐1 activity (Figure 3D). Overall, these findings suggest that HOTAIRM1 positively regulates osteogenic differentiation of MSCs, at least in part, through JNK/c‐Jun signalling pathway.

### 3.4 HOTAIRM1 positively regulates RUNX2 expression in a c‐Jun–mediated acetylation manner

**Figure 4:**
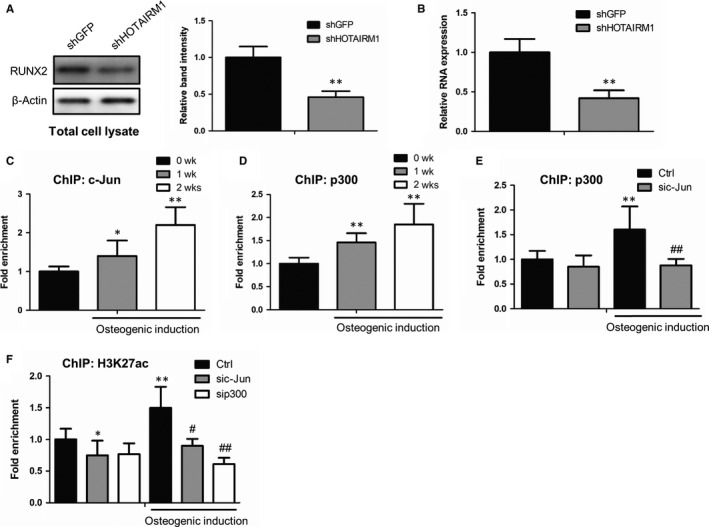
HOTAIRM1 regulates RUNX2 expression in a c‐Jun–mediated H3K27 acetylation mechanism. A, Western blot analysis showing protein expression levels of RUNX2 in MenSCs, with or without HOTAIRM1 depletion, followed by ImageJ software quantification. B, RNA expressions of RUNX2 in MenSCs, with or without HOTAIRM1 knockdown, were determined by RT‐qPCR assay. C, The binding affinity of c‐Jun with RUNX2 promoter region after osteogenic induction for 1 and 2 wk, respectively, was examined using anti‐c‐Jun ChIP assays. D, The distributions of p300 at RUNX2 promoter regions in MenSCs with osteogenic medium treatment for 1 and 2 wk, respectively, were tested by ChIP assays, with an antibody against p300. E, The effect of c‐Jun on p300 recruitment to RUNX2 promoter in MenSCs with or without osteogenic medium treatment for 1 wk was measured using anti‐p300 ChIP assays after c‐Jun knockdown. F, ChIP assay analysis showing the effect of c‐Jun and p300 on acetylation status of H3 at K27 site in the presence or absence of osteogenic medium treatment for 1 wk. c‐Jun and p300 were specially silenced in MenSCs, respectively, using a lentiviral vector‐based shRNA. All results are from biological triplicates, and data shown are the mean ± SD. n = 3. *P < .05, **P < .01 vs 0 wk or shGFP or Ctrl; ^#^ *P* < .05, ^##^ *P* < .01 vs osteogenic medium‐treated shGFP or Ctrl

To investigate the detailed molecular mechanism by which HOTAIRM1 regulates osteogenic differentiation, we examined whether HOTAIRM1 modulates expression of the master transcription factor RUNX2, one of the most crucial osteogenic regulators. Both RT‐qPCR and Western blot assays demonstrated that attenuation of HOTAIRM1 significantly repressed RUNX2 expression (Figure 4A,B). Next, we sought to illustrate how does HOTAIRM1 regulate expression of RUNX2. As is previously reported, RUNX2 can be regulated by the transcription factor c‐Jun on one hand.^27^ On the other hand, RUNX2 expression can be epigenetically activated by histone 3 acetylation at K27 site (H3K27ac).^28^ Combined with the above data that HOTAIRM1 mediated the activity of JNK/c‐jun signalling, we have been suggested that HOTAIRM1 regulates RUNX2 expression through a c‐Jun–mediated acetylation manner. To prove this hypothesis, we first employed chromatin immunoprecipitation (ChIP) assay to observe dynamic changes in c‐Jun occupancies at promoter region of RUNX2 after osteogenic induction. As a consequence, anti–c‐Jun ChIP result showed that distribution of c‐Jun at RUNX2 promoter is markedly enriched when exposed to osteogenic medium treatment (Figure 4C). Meanwhile, we assayed the H3K27ac‐modified acetyltransferase p300 binding affinity with RUNX2 promoter. Intriguingly, p300 binding ability is also increased after osteogenic induction (Figure 4D). Next, we ask whether p300 recruitment to RUNX2 promoter depends on the transcription factor c‐Jun. To test this notion, we performed anti‐p300 ChIP assays, with or without c‐Jun knockdown, to determine the effect of c‐Jun on p300 chromatin recruitment. Consequently, p300 distribution at RUNX2 promoter was remarkably suppressed after c‐Jun inhibition (Figure 4E). Furthermore, the c‐Jun–mediated H3K27 acetylation status at RUNX2 promoter was parallelly validated by anti‐H3K27ac ChIP assays after c‐Jun and p300 depletion, respectively (Figure 4F). In summary, these results suggest that HOTAIRM1 regulates RUNX2 expression in a c‐Jun–mediated acetylation manner.

## 4 DISCUSSION

HOTAIRM1 is a previously documented lncRNA, functionally involved in multiple cancers. However, the regulatory role of HOTAIRM1 in cell lineage commitment is almost unknown until now. In the current study, we first identify HOTAIRM1 as a critical activator of osteogenic differentiation of menstrual blood‐derived mesenchymal stem cells (MenSCs) and umbilical cord mesenchymal stem cells (UCMSC). shRNA‐mediated HOTAIRM1 knockdown in both of the two cell lines markedly repressed the calcium deposition and the activity of alkaline phosphatase, suggesting a critical inhibitory role in osteogenesis. Consistently, enforced overexpression of HOTAIRM1 significantly augmented osteogenic capacity of these mesenchymal stem cells. Overall, these results provide solid evidence for the potential regulatory role of HOTAIRM1 during osteogenic differentiation.

JNK/AP‐1 is widely accepted as a crucial signalling in the regulation of osteogenesis. As is reported, JNK/MAPK could act as a molecular switch to balance the osteoblast and adipocyte lineage differentiation,^29^ and JNK activation is able to enhance the osteogenic differentiation,^30^, ^31^, ^32^ suggesting the functional importance of JNK/MAPK signalling pathway in osteogenesis. Here, we observed a significant co‐relation of HOTAIRM1 with JNK/AP‐1 signalling using mRNA sequencing approach. More importantly, attenuation of HOTAIRM1 significantly restrains the activity of JNK and its downstream regulator c‐Jun. These observations suggest that HOTAIRM1 positively regulates osteogenic differentiation, at least in part, through JNK/AP‐1 signalling. Meanwhile, we identify HOTAIRM1 as a crucial regulator to mediate the activity of JNK/AP‐1 signalling.

**Figure 5:**
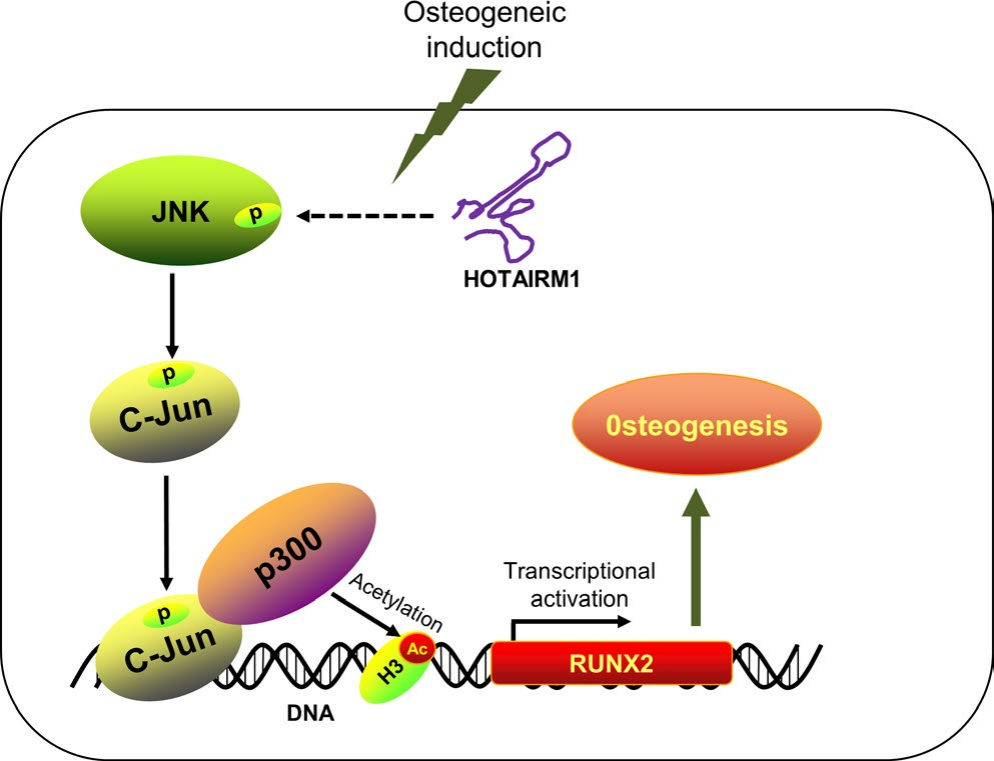
Schematic representation of the molecular basis by which HOTAIRM1 regulates osteogenic differentiation. Upon osteogenic induction, expression of HOTAIRM1 is induced and then activates the phosphorylation status of MAPK/JNK. Subsequently, the activated JNK signalling phosphorylates the transcription factor c‐Jun and help recruit c‐Jun to RUNX2 promoter region, therefore co‐activating p300‐mediated acetylation of histone 3 at K27 site, epigenetically stimulating RUNX2 gene transcription

As a major regulator of AP‐1 transcription factor family, c‐Jun is previously found to be involved in regulating the expression of RUNX2,^27^ a critical master transcription factor of osteogenesis. However, the detailed molecular basis is still unclear. In this study, we discovered that c‐Jun can serve as a partner of p300, helping recruit p300 to RUNX2 promoter region, therefore co‐activating p300‐mediated acetylation of histone 3 at K27 site and subsequent RUNX2 gene transcription, finally inducing osteogenic differentiation (Figure 5). More importantly, Loss of HOTAIRM1 can markedly inhibit the expression of RUNX2. Collectively, these findings offer us a new insight into understanding the important biological function of HOTAIRM1 in osteogenic regulation, and we conclude that HOTAIRM1 may become a promising molecular target for bone tissue repair and regeneration engineering in the near future.

## CONFLICT OF INTEREST

The authors declare that there is no conflict of interest.

## AUTHOR CONTRIBUTIONS

Xiaoyu Fu conceived and designed the project. Lei Fu and Shifang Peng performed most of the experiments. Yi Ouyang contributed to data analysis. Wanfeng Wu and Shifang Peng performed statistical analysis. Lei Fu wrote the draft of the manuscript. Xiaoyu Fu and Deming Tan revised the manuscript for important intellectual content. All authors contributed to discussions.

## ETHICAL APPROVAL

All the MenSCs and UCMSC were obtained with the informed consent of the donors. All experiments in this manuscript meet the ‘Declaration of Helsinki’ and were approved by Ethics Committee of the Central South University.

## Data Availability

The data that support the findings of this study are available from the corresponding author upon reasonable request.
